# Influence of Wobble-Based Scanning Strategy on Surface Morphology of Laser Powder Bed-Fabricated Permalloy

**DOI:** 10.3390/ma16052062

**Published:** 2023-03-02

**Authors:** Ta-Yu Huang, Chung-Wei Cheng, An-Chen Lee, Tsung-Wei Chang, Mi-Ching Tsai

**Affiliations:** 1 Department of Mechanical Engineering, National Yang Ming Chiao Tung University, No. 1001, Ta Hsueh Road, Hsinchu 300, Taiwan; 2 Department of Mechanical Engineering, National Cheng Kung University, Tainan 701, Taiwan

**Keywords:** laser powder bed fusion, scanning strategy, surface roughness, periodic surface structure

## Abstract

Surface roughness quality is still a significant problem in the laser powder bed fusion (LPBF) process. This study proposes a wobble-based scanning strategy to improve the insufficiencies of the traditional scanning strategy with regard to surface roughness. A laboratory LPBF system with a self-developed controller was used to fabricate Permalloy (Fe-79Ni-4Mo) with two scanning methods: traditional line scanning (LS) and the proposed wobble-based scanning (WBS). This study investigates the influences of these two scanning strategies on porosity and surface roughness. The results imply that WBS can maintain higher surface accuracy than LS, and the surface roughness can be reduced by about 45%. Furthermore, WBS can produce periodic surface structures arranged in fish scales or parallelograms with appropriate parameters.

## 1. Introduction

The laser powder bed fusion (LPBF) or selective laser melting (SLM) method uses a powder-spreading device to evenly distribute powder on a base plate that can be lifted vertically and control each layer’s thickness. Then, a focused laser beam is used to selectively melt the 2D sliced surface, the powder bed is lowered to allow new powder to be deposited on top, and the above process is repeated until the workpiece is manufactured.

In the LPBF process, due to the rapid heating and cooling of material during the melting process, this process results in a large temperature gradient, which significantly impacts the shape of the melting pool trajectory and the mechanical properties of the workpiece [1,2,3]. Various methods have been developed to manage the temperature gradient in LPBF, including optimization of process parameters, scanning strategy, and the use of support structures to help manage thermal stresses during the printing process. Among them, the scanning strategy can effectively disperse thermal stress [2,4] and is a currently used method. However, during the LPBF process, the workpiece still has poor surface roughness due to the thermal effect generated by the laser. The traditional solutions are mostly executed through post-processing methods (milling, peening, laser, etc.), which can significantly increase the processing time [5].

Alfieri et al. [6] found that linear and oscillating scanning laser polishing methods can reduce surface roughness. Meanwhile, Liu et al. [7] found that for repeated scanning patterns with lower laser energy between layers, the surface roughness can be reduced from 13.3 μm down to 9.9 μm. Yasa et al. [8] found that laser remelting can effectively improve the surface quality and increase the density of workpieces to nearly 100%. Although the laser post-processing method can reduce surface roughness, it leads to a longer process time because the LPBF parts have to undergo an additional remelting step.

Although laser WBS technology has been evidenced to improve process quality in welding various metals [9,10,11,12], few relevant studies have attempted to apply WBS technology in an LPBF system. Matthias et al. [13] investigated the influence of various WBSs on the distribution of laser energy density. Yang et al. [14] used a circular WBS in LPBF technology to manufacture 718 nickel-based alloys and found that this scanning strategy can effectively improve accuracy and increase material density to 99.95%. Islam et al. [15] found that a WBS could improve the surface morphology of LPBF parts. However, a fixed swing frequency for wobble scanning was used for the LPBF [13], which resulted in the swing overlap ratio of the wobble pattern varying with scanning speed and swing width.

Therefore, this study proposes a WBS with a specified wobble swing overlap rate and investigates the effects of scanning velocity, swing width, and wobble swing overlap rate on surface topography. In addition, a comparison between conventional linear scanning and the proposed WBS was conducted. Moreover, the swing overlap rate was used as a control parameter to control the characteristics of the periodic surface structure fabricated using the WBS process.

## 2. Experiment and Method

### 2.1. Experiment

A self-developed laboratory LPBF system comprising a laser source, beam delivery module, powder laying, and a chamber was used in this research [16]. The laser beam irradiated through a laser galvanometer scanner (intelliSCAN 20, Scanlab, Puchheim, Germany) and an f-theta lens (focal length 310 mm); the focal spot on the powder bed was about 60 μm. The control algorithm for the LPBF system was developed under the CoDeSys environment. The LPBF system transmitted data via EtherCAT with a 0.1 ms cycle time, thus realizing the process control, linear scanning (LS), and wobble-based scanning (WBS) strategies. In the LPBF process, Permalloy (Fe-79Ni-4Mo) was deposited with a 20 μm layer thickness on a base plate (S45C) substrate. The LPBF process was performed in a chamber filled with protective gas (nitrogen) with a uniform flow rate on the powder surface. The oxygen concentration was controlled to less than 3000 ppm to avoid oxidation during processing.

After LPBF processing, the fabricated sample was removed from the base plate via wire cutting for analysis. An optical microscope (OM, Axioskop 40, Zeiss, Jena, Germany) was used to measure the surface morphology and cross-section of the fabricated sample. The specimens were ground with sandpaper (No. 80-2500) and finally polished with a 1 μm alumina polishing solution. Density was analyzed in the cross-sectional images using ImageJ software. The process was as follows: convert pores into black pixels, convert complete fusion areas into white pixels, use the plug-in program Color_Counter.class for the color calculation to count the ratio of black and white pixels in the screen, and then calculate density. Surface roughness was measured using a laser confocal microscope (VK-X3000, Keyence, Osaka, Japan).

Focusing on the influence of WBS on surface roughness, the line roughness measurement direction in this study was parallel to the laser scanning direction. Then, the arithmetic mean deviation based on the measured data was calculated to obtain the surface roughness Ra value.

### 2.2. Wobble-Based Scanning

**Figure 1:**
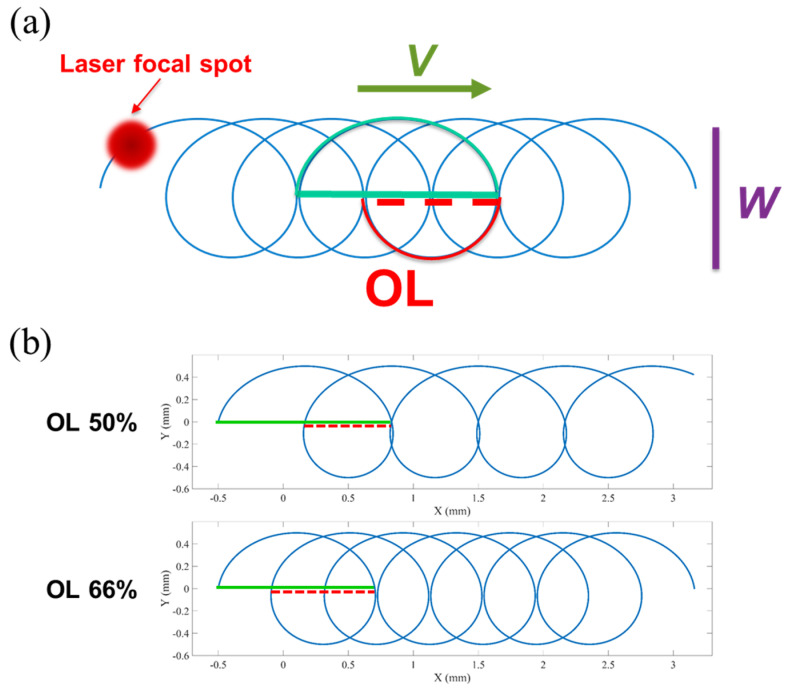
(**a**) A schematic diagram of the WBS and (**b**) examples of the trajectory of different OLs, 50% and 66%.

The schematic diagram of the WBS is shown in Figure 1a, where *W* is the swing width, *V* is the linear scanning speed along the WBS direction, and OL is the swing overlap rate. The solid green line is the displacement distance when the track swings forward, and the dotted red line is the displacement distance when the track swings back. The swing overlap rate OL is defined as the length of the dotted red line divided by the length of the solid green line. Figure 1b shows the example of the trajectory of different OLs of 50% and 66%.

The parametric equation of the WBS trajectory used in this study is defined below:

(1) $$x\left(u\right)=-\frac{W}{2}\cos\left(\omega u\right)+Vu$$

(2) $$y\left(u\right)=\frac{W}{2}\sin\left(\omega u\right)$$

where x(u) and y(u) are the positions of the laser galvanometer scanner; *W* and *V* are defined as shown in Figure 1a; u is the parameter; ω=2πf is the angular frequency (rad/s); and *f* is the swing frequency (Hz).

In order to prevent the swing trajectory from being affected by the settings of the swing width *W* and the scanning speed *V*, this study explores the relationship between WBS parameters OL, *W*, *V*, and swing frequency *f*. In particular, this study discusses the position difference of the trajectory in the x direction (Equation (1)). The length of the dotted red line in Figure 1a is the length of the solid green line multiplied by the OL. The solid line is the distance from the initial position of the trajectory to the position of 1/2 cycle. Thus, the length of this segment for the displacement distance *u* = 0 to $\frac{1}{2f}$ is equal to the distance of the trajectory from the ½-period position to the 1-period position *u*= $\frac{1}{2f}$ to $\frac{1}{f}$. The following relationship is obtained:

(3) $$\mathrm{OL}\times \left\{x\left(\frac{1}{2f}\right)-x\left(0\right)\right\}=x\left(\frac{1}{2f}\right)-x\left(\frac{1}{f}\right)$$

From Equations (1) and (2), Equation (3) is expressed in the form:

(4) $$\begin{array}{cc} \mathrm{OL}\times \{[-\frac{W}{2}\cos & \left(\frac{2\pi f}{2f}\right)+\frac{V}{2f}]-\left[-\frac{W}{2}\right]\}\\ & =\left[-\frac{W}{2}\cos\left(\frac{2\pi f}{2f}\right)+\frac{V}{2f}\right]-\left[-\frac{W}{2}\cos\left(\frac{2\pi f}{f}\right)+\frac{V}{f}\right] \end{array}$$

After simplifying the relation (4), we can obtain the following:

(5) $$OL\left(W+\frac{V}{2f}\right)=W-\frac{V}{2f}$$

It is further known that:

(6) $$f=\frac{\left(1+OL\right)V}{2\left(1-OL\right)W}$$

In this study, given *OL*, *W*, and *V*, the swing frequency *f* in Equations (1) and (2) can be obtained according to (6). The total processing time of the single-line trajectory was calculated according to the linear scanning speed *V*, and the tangential velocity along the curve was calculated from the total length of the trajectory path. For a constant tangential velocity along the WBS path, the laser position command for each interruption time was obtained through parameter interpolation calculation [17].

In this study, the contour error of the trajectory was calculated by comparing the commanded trajectory with the actual scanning position of the laser galvanometer. The radius of the laser spot was used as the upper limit of the allowable contour error to confirm the controllability of the laser scanning galvanometer. The laser focus spot diameter of this experimental system was about 60 μm; hence, 30 μm was used as the allowable value of the contour error in the algorithm. When the contour error was more than 30 μm, the algorithm marked the position, and when the number of marked points accounted for more than 5% of the trajectory points, this parameter was considered not applicable.

For the WBS and LS scanning strategies, the distance between adjacent scanning paths was defined as hatch distance (HD), and a unidirectional scanning pattern filled the area. In both scanning strategies, the SD was rotated by 90° after every single layer.

## 3. Results and Discussion

### 3.1. Single-Layer Experiments

**Figure 2:**
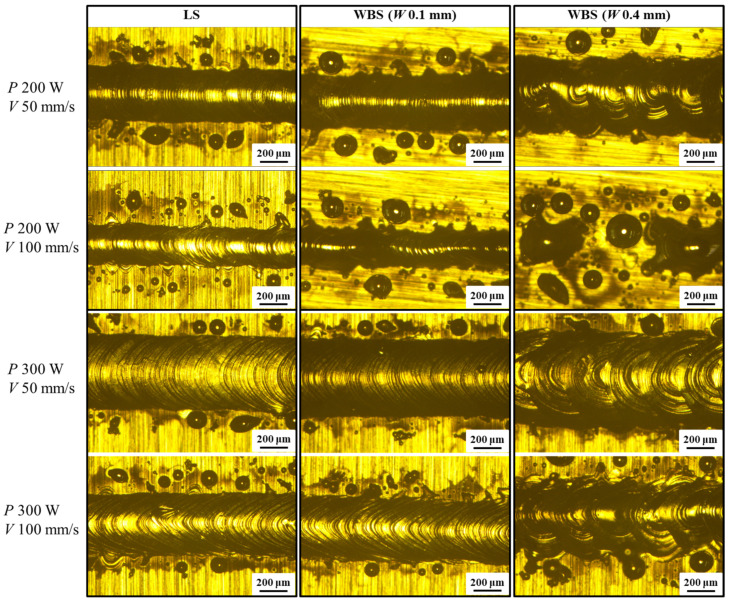
Line track melting morphology of LS and WBS under different parameters.

Figure 2 presents the surface morphology of the single melt tracks on the substrate made by LS and WBS scanning strategies under different parameters. The laser power (200, 300 W) and scanning speed (50, 100 mm/s) were used to make LS and WBS single-line traces. A swing overlap rate OL of 50% and a swing width *W* of 0.1 and 0.4 mm were used for WBS. As shown in Figure 2, when the laser power was 200 W and the scanning speed was 50 mm/s, both LS and WBS completely and stably melted the powder and formed a continuous trajectory. When the radiation power was 200 W and the scanning speed was 100 mm/s, the WBS trajectory with *W* 0.1 and 0.4 turned from a continuous form into a discontinuous type. This can be explained by Plateau–Rayleigh instability: the melt track shrinks excessively during solidification, and a drop-shaped broken track is formed [18]. With a laser power of 300 W, the melting tracks of LS and WBS were almost complete. When *W* was 0.4 mm and the scanning speed of WBS was 50 mm/s, an apparent swing phenomenon could be observed under different powers.

**Figure 3:**
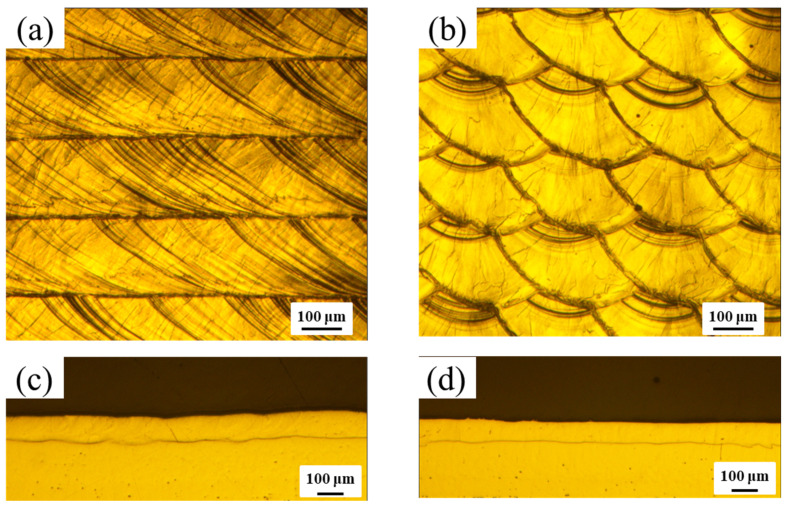
Single-layer surface morphologies of (**a**) LS and (**b**) WBS and (**c**,**d**) the cross-sectional morphologies of (**a**,**b**), respectively.

For single-layer area fabrication via LS and WBS, 300 W laser power, 50 mm/s scanning speed, and HD 200 μm were employed. The OL was 50%, and *W* was 0.4 mm for WBS. The surface and cross-sectional OM morphologies (perpendicular to the laser scanning direction) images are shown in Figure 3. From Figure 3a, it can be found that when using LS, the melt tracks are parallel to the scanning direction. However, due to the influence of the molten pool swing of the WBS, the melt tracks can form different shapes, as shown in Figure 3b. Figure 3d shows that the surface of the molten pool and the remelted depth are flatter than LS, mainly due to the influence of the swing of the molten pool, making the surface flatter during the LPBF process. Yang et al. [14] used circular WBS to print 718 nickel-based alloys and found that the molten pool morphology could be transformed from inverted peaks (by LS) to rectangle shapes (by WBS), producing better formability.

**Figure 4:**
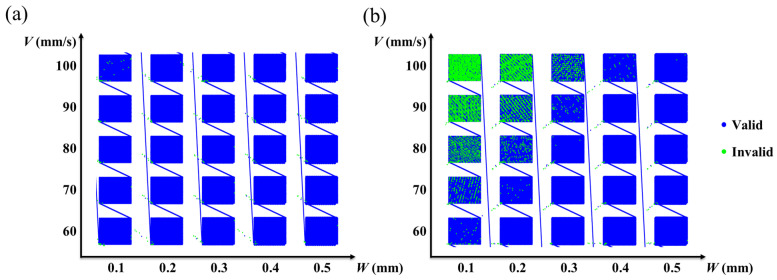
Reliable parameter judgment results (**a**) OL: 50% (**b**) OL: 66%.

For a higher-scanning-speed WBS process, contour error inspection (see Section 2.2 for details) was carried out with the parameters of swing overlap rate OL as 50% and 66%, the swing width *W* as 0.1–0.5 mm, and the scanning speed as 60–100 mm/s. The analysis results are shown in Figure 4. Note that invalid (marked by green circle) means the contour error of the trajectory point was more than 30 μm (half times the laser focus spot diameter). The error point ratio is the number of invalid points to entire trajectory points. In this study, an error point ratio of more than 5% was considered not applicable to the WBS process. As shown in Figure 4b, when the *W* was 0.1 mm and the scanning speeds were 90 mm/s and 100 mm/s, the error point ratios were 8.23% and 15.55%, respectively. When the *W* was 0.2 mm and the scanning speed was 100 mm/s, the error point ratio was 11.12%. Therefore, the above parameters were judged not applicable, and other parameters in Figure 4 were judged as reliable. The calculation results of the tangential scan rate for this set of swing parameters are shown in Table 1. We found that the maximum tangent scan rate in this set of swing parameters was 764 mm/s, constrained by the bandwidth of the laser galvanometer scanner (intelliSCAN 20, Scanlab) used.

### 3.2. Multilayer Experiments

In this study, a multilayer experiment was executed by maintaining the laser power density under the following conditions: laser power of 250 W, swing overlap rate OL of 50% and 66%, swing width *W* of 0.1~0.5 mm, and scanning speed *V* of 40~80 mm/s. Then, using two kinds of hatch distance HD—100 μm and 200 μm—5 mm · 5 mm size specimens with 12 layers were fabricated. Table 2 shows the results of the density analysis. The density of LS was 99.90%, while the density of WBS was about 0.03 ~ 0.07% higher than that of LS.

**Figure 5:**
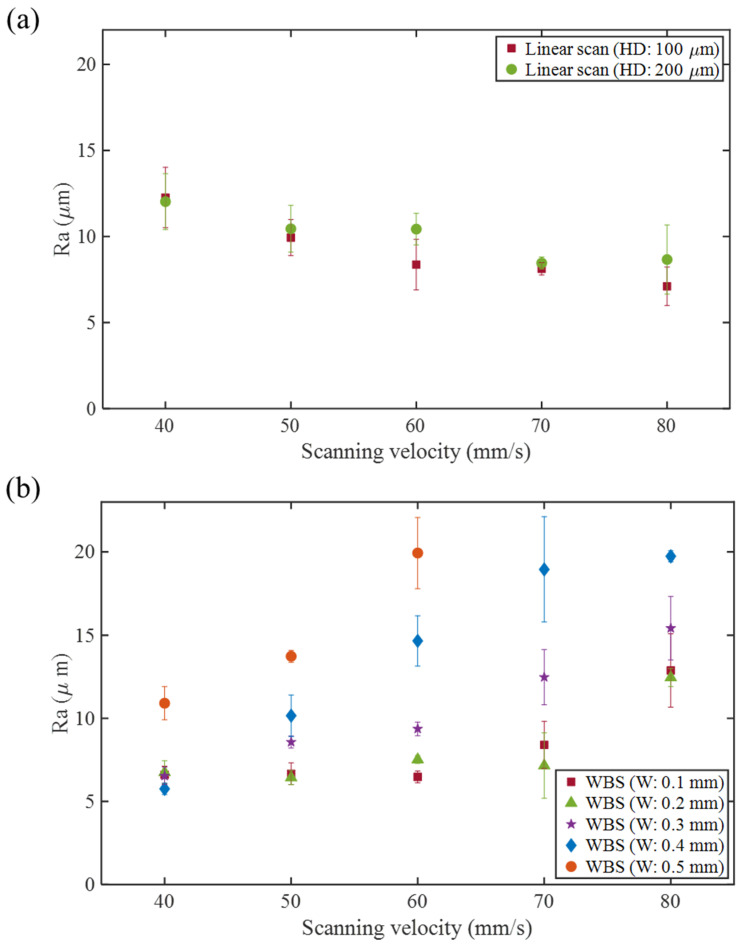
Surface roughness at different scanning speeds: (**a**) LS (HD 100, 200 μm), (**b**) WBS (HD200 μm, 66% OL, *W* 0.1~0.5 mm).

Figure 5 shows the surface roughness of LS and WBS at different scanning speeds (40–80 mm/s). It can be seen from Figure 5a that under LS, the surface roughness tended to decrease as the scanning speed *V* increased. The surface roughness Ra of the LS specimen with HD of 100 μm decreased from 12.3 μm at a scanning speed of 40 mm/s to 7.1 μm at 80 mm/s. On the other hand, the LS specimen with an HD of 200 μm decreased from 12.0 μm at a scanning speed of 40 mm/s to 8.7 μm at a scanning speed of 80 mm/s. When the scanning speed increased, the surface roughness Ra gradually decreased. When the scanning speed was slow, i.e., 40 mm/s, the irradiated time on the powder surface was prolonged, the heat-affected zone was increased, and the subsequent solidification structure had large morphology [19], which increased the roughness.

Figure 5b shows the experimental results of WBS using HD 200 μm with an OL of 66% and different *W* 0.1–0.5 mm. As the scanning speed *V* increased, the surface roughness tended to increase. Taking *W* = 0.4 mm as an example, the surface roughness Ra increased from 5.7 μm at a scanning speed of 40 mm/s to 19.7 μm at a scanning speed of 80 mm/s. When the *W* was 0.5 mm, the surface was not wholly stacked when the scanning speed was over 70 mm/s; hence, the surface roughness measurement was not performed. Although the average laser power density of LS and WBS was the same, the energy distribution formed by WBS during scanning helped reduce the maximum temperature of the molten pool. The advantages of WBS are that a rectangular molten pool shape can be obtained [14], and the temperature gradients during the process can be reduced [20]. However, when the scanning speed increases, the temperature of the molten pool gradually tends to be insufficient, which makes the molten pool unstable, eventually leading to an increase in surface roughness.

**Figure 6:**
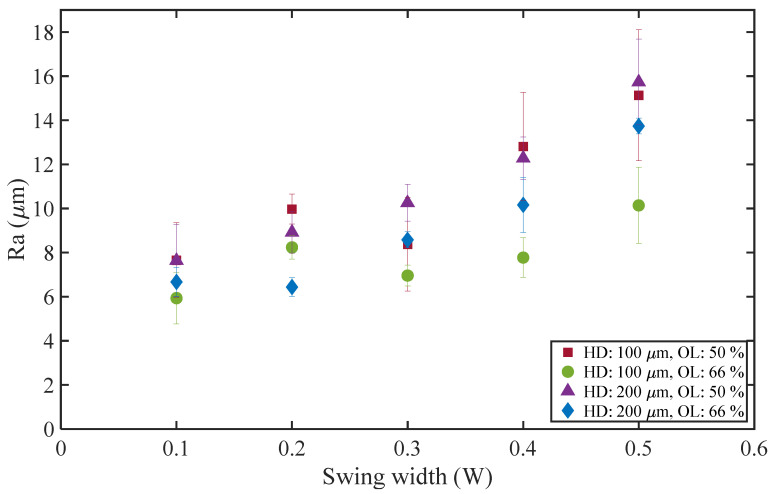
Surface roughness of WBS at different swing widths (scanning speed 50 mm/s).

Figure 6 shows the surface roughness of WBS at different swing widths *W* where the scanning speed is 50 mm/s. It can be seen that as the *W* increases, the surface roughness tends to increase. For example, the surface roughness increases from 5.9 to 7.6 μm when the *W* is 0.1 mm to 10.1 to 15.7 μm when the *W* is 0.5 mm. It is speculated that this phenomenon is due to the fact that the swing width is too large, which makes the melt pool shape change from a single straight line to a melt pool shape with obvious swing track characteristics (see Figure 2).

The surface roughness of HD 100 μm decreased in the range of 0.2–0.3 mm of *W* and then gradually increased. It was speculated that the equivalent laser power density generated by overlapping tracks was too high at HD 100 μm; thus, when *W* was 0.1–0.2 mm, the surface roughness increased sharply. However, when *W* increased to 0.3 mm, due to the large range of laser energy distribution, the shape of the molten pool changed from a single straight line to a swing track feature. Therefore, the surface roughness decreased slightly and gradually increased with the swing width.

**Figure 7:**
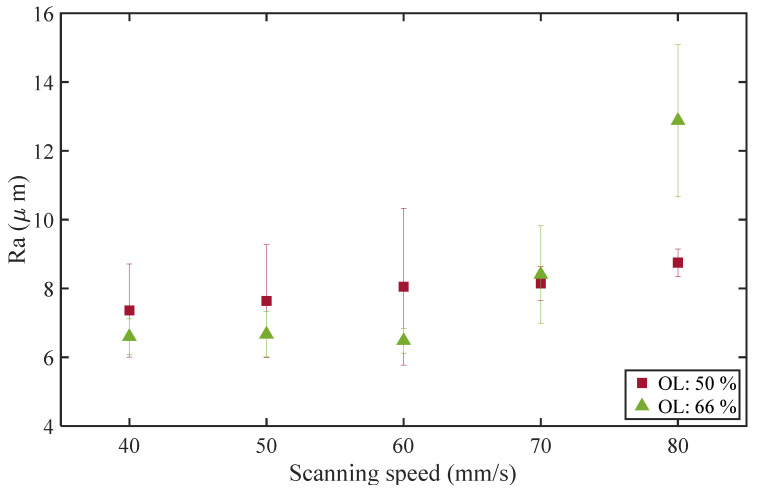
Surface roughness of WBS at different swing overlap ratios and scanning speeds.

Figure 7 shows the surface roughness of WBS at an OL of 50% and 66% and a scanning speed of 40–80 mm/s. The *W* is 0.1 mm and HD is 200 μm. At the same *W*, the surface roughness at OL 66% is roughly smaller than that at 50%, except for the scanning speed of 80 mm/s. At a scanning speed of 40 mm/s, the roughnesses of WBS at OLs of 50% and 66% are about 7.4 and 6.6 μm, respectively. On the other hand, the roughness of LS is about 12.0 μm (Figure 5a). Compared with LS, WBS can reduce surface roughness by about 38% and 45%.

It is speculated that WBS at better parameters can homogenize laser energy and reduce the highest energy density distribution [21]. However, at a scanning speed of 80 mm/s, the high OL destabilizes the molten pool, resulting in high surface roughness. Alfieri et al. [6] used WBS to post-process metal additive-manufactured parts and found a general rule: increasing wobble frequency for a given wobble swing width decreased surface roughness. In this study, Equation (5) describes the relationship between *OL*, *W*, *V*, and *f*. The OL becomes more significant when *W* and *V* are fixed due to increased *f*. Accordingly, the surface roughness at OL 66% was roughly smaller than that at 50%.

**Figure 8:**
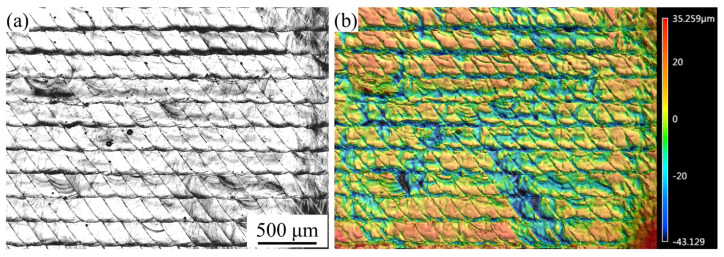
Surface topography: (**a**) OM, (**b**) 3D topography.

**Figure 9:**
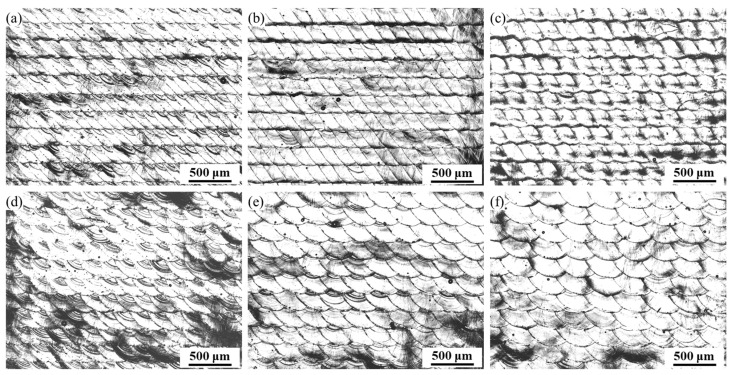
Surface topography: OL 66%, *W*: (**a**) 0.3 mm, (**b**) 0.4 mm, (**c**) 0.5 mm, OL 50%, *W*: (**d**) 0.3 mm, (**e**) 0.4 mm, (**f**) 0.5 mm.

As shown in Figure 6, although the WBS uses a larger swing width to increase the roughness, it can also form periodic surface topography features on the surface at the same time. In this study, the HD was 200 μm, and the WBS test piece was used for surface morphology analysis, as shown in Figure 8. From the 3D topography (Figure 8b), it can be seen that the WBS leaves a periodic raised structure on the surface. Figure 9 presents the OM diagram of the *W* of 0.3~0.5 mm and different OLs (50% and 66%). Figure 9a–c shows results for the OL of 66%, while Figure 9d–f shows results for the OL of 50%. It can be observed that the morphology of the OL of 66% is closer to the parallelogram arrangement, while the morphology of the OL of 50% is the fan-shaped arrangement. As the *W* increases from 0.3 mm to 0.5 mm, the width of the 66% OL topography features increases from 119 μm to 205 μm, while the width of the 50% OL topography features increases from 211 μm to 333 μm.

In order to confirm whether the periodic surface structure generated using WBS is controllable, this research compared the output data of the program with the actual surface topography under different swing overlap ratios and swing widths. The measurement object was a test piece with 0–66% OL and 0.2–0.5 mm *W*. The error percentage between the actual morphology characteristics and the program point data characteristics after the comparison and confirmation of the two morphological features is shown in Table 3. The comparison results show that the error between the two was about 6.3%, which means that the actual surface topography was a controllable parameter.

## 4. Conclusions

In this study, WBS with a specified overlap ratio in the LPBF process was successfully demonstrated and investigated. The results show that the WBS technique has potential benefits in reducing surface roughness. For example, under a swing width *W* of 0.1 mm, scanning speed of 40 mm/s, and swing overlap rate OL of 50%, WBS can reduce surface roughness by about 39% compared with LS. When the swing overlap rate OL was 66%, the surface roughness could be further reduced by about 45%. Furthermore, compared with LS, WBS can fabricate periodic structures. When using an OL of 50%, a fish-scale periodic structure can be obtained on the surface; using an OL of 66%, a periodic structure of a parallelogram arrangement can be left on the surface.

## Figures and Tables

**Table 1:** Tangential velocity (mm/s) calculation results.

OL (%)	*V* (mm/s)	*W* (mm)				
		0.1	0.2	0.3	0.4	0.5
66	100	764	759	759	759	733
	90	688	683	683	683	660
	80	612	607	607	607	587
	70	535	531	531	531	513
	60	459	455	455	455	440
50	100	472	472	463	462	454
	90	425	424	417	416	408
	80	378	377	370	370	363
	70	330	330	324	323	318
	60	283	283	278	278	272

**Table 2:** Density analysis of test pieces.

OL (%)	*W* (mm)			Linear Scan	
	0.1	0.3	0.5	99.90	
66	99.97	99.96	99.79		
50	99.93	99.95	99.95		(%)

**Table 3:** Surface topography error.

Overlap (%)		W (mm)			
	0.2	0.3	0.4	0.5	
66	6.3	0.6	0.7	2.6	
50	0.8	5.4	1.8	0.8	(%)
